# The Community Pharmacist: Perceived Barriers and Patient-Centered Care Communication

**DOI:** 10.3390/ijerph17020536

**Published:** 2020-01-15

**Authors:** Maria Laura Ilardo, Antonio Speciale

**Affiliations:** 1 Department of Biomedical Sciences and Public Health, Polytechnic University of Marche, 60020 Ancona, Italy; m.l.ilardo@pm.univpm.it; 2 Department of Chemical, Biological, Pharmaceutical and Environmental Sciences, University of Messina, 98168 Messina, Italy

**Keywords:** effective communication, community pharmacy, modern pharmaceutical services, on-line pharmacy, health literacy, pharmacist’s re-professionalization, communication skills improvement, efficient consultancy service

## Abstract

Nowadays, the classic perception of the pharmaceutical profession in community pharmacies is facing worldwide extinction due to many factors. Among the numerous factors, online pharmacies are increasingly gaining ground thanks to their ability to facilitate customer demand. Nevertheless, they are endangering “face-to-face” contact, affecting the building of customer loyalty based on direct “human” interaction, and consequently reducing pharmacists to mere commercial figures. Patient-centered care communication is emphasized as the essential element to build a solid and appropriate interpersonal relationship with the patient, to make the consultancy process effective, and to strengthen the pharmacist’s professionalism in community pharmacy. This paper presents a narrative review of existing literature with the first aim of pinpointing the factors affecting pharmacy professional practice, and secondly, of how to improve patient-centered communication skills. A more widespread introduction of in-depth study and practice of behavioral, communication, educational, and sociological methodologies and techniques would allow for the development of more effective skills used for providing an efficient consultancy service, improving the capacity of future professionals to approach public relations.

## 1. Introduction

The progress of scientific and technological knowledge, the socio-economic and political changes, the demographic growth and the development of National Health Systems, and the birth of clinical pharmacy and pharmaceutical care have all contributed to the evolution of the pharmaceutical profession, which began to expand into other sectors: community pharmacy, hospital pharmacy, pharmaceutical industry, regulatory control, drug management, and academic activity [1,2,3,4,5,6]. Among these, community pharmacists have been defined as “the first port of call” among the professionals in the health sector thanks to their easy accessibility to the public [7,8]. They can also be described as “primary care pharmacists,” thus recognizing their contribution in delivering primary health care services, including management of chronic conditions (e.g., hypertension, treatment of minor ailments, administration of vaccinations) [9,10,11,12].

The pharmacist acts as an intermediary between the doctor and the patient by providing both medicines and free medical advice without the need for an appointment. Although pharmacists can be the first point of contact for some healthcare consumers, they are a relatively underutilized resource and almost “invisible” in recent health care policies [13,14,15].

In order to enhance their profession, pharmacists have begun to reinvent themselves by finding solutions to extend their role in community pharmacy. In general, they focused on establishing a closer relationship with the patient and enhancing their professionalism by self-assuming the great responsibility of providing appropriate advice (decision-making power) [3].

With the aim of preventing, protecting, and promoting the patient’s health, the pharmacist has to give clear and easily understandable information about the correct use of a drug and its possible contraindications so that the patient gets the maximum benefit from it (problem-solving professional) [3]. For example, as medication experts prepared to dispense opioid prescriptions, they can play a key role in contributing to safe opioid use among patients [16] by educating them on risks associated with opioid use (e.g., disposal of medication, the consequences of sharing medications with another person) [17]. Although pharmacists are prepared to provide these services, current literature documents that these opportunities are far from being fully realized [18]. Community pharmacists may also play an important role in increasing medication adherence, thus contributing to decreasing morbidity, mortality, and health care costs [19]. In this regard, Conn and Ruppar [20] revealed that the most effective interventions were delivered face-to-face and administered directly to patients, and that the pharmacist intervention is more effective than those delivered by other healthcare professionals.

Despite all of these attempts to strengthen the profession through a new vision more inclined to customer care, today the perception of the pharmaceutical profession is increasingly at risk. It is mainly undermined by health policies, by the growing presence of drugs in supermarkets, by the increase in multiple-shop pharmacies, and by the impact of Information Technology on society. The growing use of online and mail-order pharmacies has certainly facilitated the customers but has also deprived them of the physical encounter with professionals in pharmacy. Thus, by resorting to these means, personal communication between the pharmacist and the patient has been reduced or has even disappeared [2,3,21,22]. The pharmacist in community pharmacy is thus penalized, his/her skills are considered obsolete (“deskilling”), and the public perceives him/her as a mere supplier of pre-packaged medicines (de-professionalization process) [3,22,23,24,25]. In this regard Hindi et al. [26] found that in the UK the pharmacy is seen as a medicine supply shop by 48.3% of people, as a place to purchase medicines by 22%, and a place to purchase non-medicinal products by 17.7%. Therefore, only 1% of people are prompted to go to a community pharmacy because of “the trust in the pharmacist” [26]. In fact, in order to face this downward path, the pharmacist must aim to establish a loyalty-based relationship to strengthen his position and competitiveness on the market (“re-professionalization” process) [27,28].

As Cipolle et al. state, “Care means communication. Quality care means quality communication” [29]. Since one of the key roles of the pharmacist in community pharmacy is focused on counseling, communication is essential in order to fulfill the primary ethical duty, namely, to protect and improve the health of each individual patient. Communication is therefore the key element for making the consultancy process effective and to strengthen the future of the pharmacist profession.

Given that the use of effective communication skills is considered essential to providing adequate assistance and advice to patients, the study and practical application of behavioral methods and communication techniques would permit pharmacists to establish a productive dialogue with the patient and thus increase social relations with the public.

This paper presents a narrative review of existing literature with the aim of giving an analytical and reflective reading on the pharmacist’s role in modern society. The research was performed by pinpointing factors affecting the pharmacy professional practice and by identifying studies focused on how to improve patient-centered communication skills and health literacy. On the basis of the literature consulted and the practical cases analyzed, we underline the importance of the quality and the impact of patient-pharmacist communication. This was done with the specific purpose of exploring the possibility of translating it into a more widespread introduction of in-depth academic study and professional training of behavioral, communication, educational, and sociological methodologies and techniques. These would potentially allow a common improvement in the pharmacy practice through the development of specific and effective communication skills in order to provide an even more efficient consultancy service and to enhance the future community pharmacists’ capacity to approach public relations.

## 2. Community Pharmacists’ Perceived Barriers to Their Professional Status and Practice

In general, as Hughes [30] and Friedson [31] claimed, a profession requires the possession of a legal license and is self-regulated, thus establishing its own specific training, services, and set of skills. In addition, a profession almost always has precise relationships with both the public and the State government. It should act for the public interest and, in order to fulfill its own professional ethical duty, it should be guaranteed and ensured with a professional monopolist-status [3,24,32].

Even if they are often underestimated, the pharmacist, far from being a mere “trader,” is a health professional with a specialized knowledge in science who provides the public with services aiming to protect patients’ health and to ensure a correct, effective, and rational use of drugs [14,33,34]. In addition to the often quite diverse State legislation (at least in the EU), the pharmaceutical practice is directed and regulated by its own organizations and disciplinary bodies (e.g., in Europe, the Royal Pharmaceutical Society of Great Britain (RPSGB), the Federal Union of German Associations of Pharmacists (ABDA), and the Federazione degli Ordini dei Farmacisti Italiani, (FOFI)) which direct, regulate, and strengthen the profession and professional training [3,15,24,32,34].

This professional frame is endangered first and foremost by incongruent public intervention when policymakers, instead of considering pharmacy as a branch of the health sector and the pharmacist as a health professional, tend to regulate it as a business company like any other commercial enterprise, thus ignoring its specific role in society [3,24]. An example of such policies is represented by the generalized drug liberalization which certainly led to a rise in terms of the purchase and sale of over-the-counter drugs (OTC), but that also led patients, no longer forced to request medical and pharmaceutical advice, to acquire greater autonomy in terms of self-medication [3]. It must be said that, unlike in the past, today patients generally feel to be better informed in medical-scientific topics, considering their knowledge to be almost expert-like [35].

Consequently, also because of the misinterpretation of internet information, the greatest risk is that most people often operate a risk-benefit assessment with the high probability of incurring serious side effects. In particular, the elderly are at a higher risk because they are frequently not well informed about the appropriate dosing of Over the Counter OTC medications and/or their interaction with other drugs. Moreover, frequently health providers do not know which OTC drugs their patients are taking [36]. All this may lead to therapy duplication and dangerous overdosing, and there are no available interventions helping in the prevention of OTC medication misuse. Thus, the detection and prevention of potential drug-related problems through the pharmacist intervention are considered key to reducing adverse drug events (ADEs) [37,38].

As mentioned above, another factor negatively affecting community pharmacies, is, with few exceptions (e.g., France), the progressive loss of monopoly in drug dispensing [39]. Liberalization certainly brings advantages in terms of purchasing prices for consumers, however, dealing with a delicate matter such as drug dispensing and medical advice, it could be safer to limit the authorized suppliers, hence creating a professional monopoly held by specialized professionals [32].

Nonetheless, pharmacists are in competition with other market players who implement drug distribution based on mere economic purposes (non-pharmacy outlets, e.g., supermarkets in Canada and USA) [24,39]. As pointed out by Taylor [3], no supervision or consultancy of “experts” is needed at the moment of purchasing, thus patients, who become mere consumers, buy medicines as if they were common commercial products. Other cases are instead represented by de facto community pharmacies where, due to the internal organization (e.g., employment of less qualified personnel) and product placement (e.g., self-service), the perception is that the professional intervention and the client/patient-pharmacist relationship, except in the case of prescription-only medicines (POM) (e.g., antibiotics), is reduced to the one between a consumer and a shop cashier. This interpersonal relationship is endangered even more by the ever-increasing presence on the market of pharmaceutical chains (e.g., Czech Republic) mainly due to vertical (e.g., wholesaler takeover) or horizontal (e.g., competitor takeover) integration [40]. Even though, at least for the EU, certain national legislation (e.g., Spain, Italy, France, Greece, and Germany) explicitly limits or prohibits pharmacy chains and requires pharmacist ownership. The most critical consequence is the standardization of services that, again, is mainly based on economic and profit evaluations, and in general is a progressive loss of independence by pharmacists-owners [32,39,41]. In this regard, Taylor reports that an increasing number of pharmacies have chains and supermarkets as their owners, hence certifying a loss of professional independence (e.g., Asda, Safeway, Tesco, and Sainsbury) [2,3,32].

Another category of services endangering the profession, differing mostly in terms of delivery method, is represented by the entry (right after mail or telephone ordered [24,39]) of online community pharmacies [22]. Even though online pharmacies guarantee a clear set of advantages for the customer/patient (e.g., 24-h availability, time saving, discounted prices, cyber doctors, anonymity/privacy, access to own dispensing records, home delivery for chronic drug users, etc.), there are substantial disadvantages in terms of quality and the impact of patient-pharmacist communication, and thus also on the consultancy service [32].

First and foremost, the inability of immediate response to requests of urgent advice could lead to serious health problems for the patient, hence, for instance, the prohibition in Austria of long-distance drug sale where only physical pharmacy outlets are allowed to dispense medicines [32]. Secondly, an online consultancy service is delivered by the pharmacist only after filling out a questionnaire (usually with fees) which requires the preliminary screening of a doctor. The major consequence of this type of service (leaving aside the issue of processing personal data) is the loss in the establishment of an indispensable direct patient-pharmacist relationship (physical and verbal contact) [21,22]. Again, new technological solutions, such as the quasi-automated call centers to manage refills, are having an increasing impact in terms of employment and qualification needs for community pharmacy [15].

Considering what has been said up until now and leaving aside the objective advantages that these alternative pharmacy structures and services offer, these new trends are seen by many pharmacists and some patients as a threat to effective health care. They consider internet and mail order pharmacies as impersonal and distant “factories of prescriptions,” where an interpersonal, loyalty-based patient-pharmacist relationship is impossible. The pharmacist suffers a loss in the face-to-face dialogue with the patient, a reduction of what is his/her professional role in community pharmacy, and is penalized in his/her ability to offer efficient public services [22,23,24]. Moreover, these new solutions are substantially changing the nature of community pharmacy by “decoupling the compounding and dispensing functions of the profession of pharmacy from the medication management/cognitive services functions” [15].

**Figure 1:**
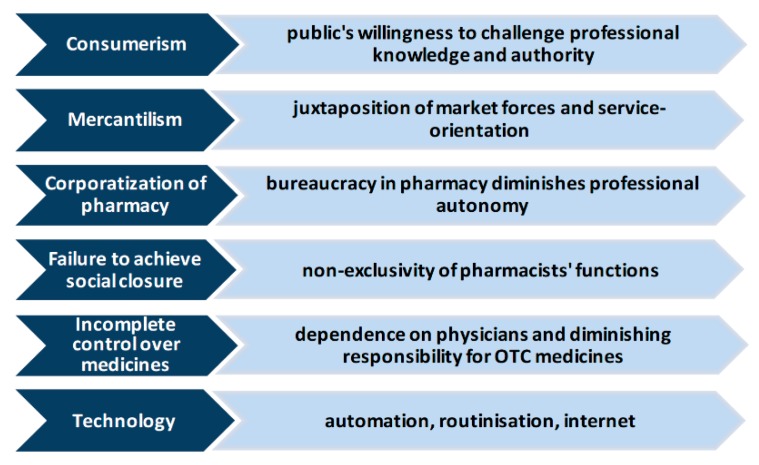
Factors undermining the pharmacy’s professional status.

In the light of what is considered in this section, it is possible to identify and highlight the factors that are undermining the professional status of the pharmacist, and they are condensed in Figure 1 [3].

## 3. Re-Professionalization Process: Communication and Patient-Centered Care

The pharmacist’s exposure to all the above-mentioned changes can be described as an example of “de-skilling.” These factors are putting the profession at a double risk: the pharmacist’s skills are considered obsolete, and the public could perceive him/her as a mere supplier of pre-packaged medicines. It is clear that the profession itself needs to be renewed in some way and a fundamental support role must be played by appropriate policies, designing better health services that can be applied by the pharmacists to each patient [15]. The community pharmacist, together with the deepening of an indispensable scientific and medical knowledge, must acquire a broader preparation in communication-related fields. This opposite process can be defined as “re-professionalization” [3]. It requires the pharmacists to strengthen the loyalty-based relationship which is the basis of the community pharmacy professional practice. More specifically, as initially enlightened by the 1997 WHO report *Preparing the Pharmacist of the Future: Curricular Development*, the pharmacist, in order to be defined as an effective communicator, must focus on building an open exchange of information and on involving patients in the treatment decision-making process [3,28,42,43].

The importance of communication and therefore the use of effective communication skills is essential to provide adequate assistance and advice to patients [27]. It all can be translated into a more productive dialogue with patients, thus an efficient patient-centered care service (PCC). Even though there is no universal agreement on its meaning, PCC can be described as “providing care that is respectful of and responsive to individual patient preferences, needs, and values, and ensuring that patient values guide all clinical decisions” [44,45,46]. In other terms, PCC is the pharmacist’s ability to recognize, understand, and manage each patient specifically before giving appropriate advice by providing cognitive pharmaceutical services (CPS) [46,47].

Furthermore, in the last few years the use of “person-centered” rather than “patient-centered” care has been preferred, which embraces much more than a person’s medical or clinical needs and preferences [48]. The guiding principles of the profession should in fact be person-focused, effective, safe, comprehensive/complete, longitudinal, collaborative, equitable, accessible, and integrated. These should constitute the core of the service provided in order for the pharmacist to guarantee the safest and most effective care possible [15].

Numerous are the contributions addressing the matter, and all of those individuate multiple dimensions framing the concept of PCC, not necessarily from the pharmacy context point of view. Among these, we refer to the seminal work of Mead and Bower, who defined five PCC dimensions which can also be applied to community pharmacy practice. Leaving aside the chosen reference literature, it must be remembered that the various PCC dimensions are to be considered correlated and dependent on one another [46,49,50]. In particular, the pharmacist must be able to: 1. understand the patient’s illness experience: social, psychological, and biomedical factors; 2. perceive the experience of each patient as unique, and conceive first of the patient as a person; 3. Promote, in as far as possible, an equitable relationship with all patients; 4. create a “therapeutic alliance” with patients in order to achieve the objectives of mutual interest; 5. develop self-awareness of the personal effects on patients [27].

The pharmacist-patient communication process is characterized by a “first acquaintance” moment during which the pharmacist must be able to: 1. perceive if the patient is subject to possible misleading influences from family members; 2. perceive if there are cultural differences regarding the conceptualization of “health” and “illness”; 3. perceive the patient’s level of knowledge about his/her health problem (people differ greatly from each other in terms of levels of medical and biological knowledge); 4. interpret the psychological characteristics of the individual, including personal motivations and objectives [27].

Therefore, it is essential that the pharmacist is able to distinguish which patient is in possession of a sufficient level of medical-scientific knowledge and which is not. This aspect is fundamental for the pharmacist’s use of medical terminology, which needs to be expressed in the most simple, accessible, and confidential way [51]. Again, in order to conduct a more effective consultancy process, the patient must be recognized as passive or active and the message must be tailored considering sex, age, and social background [3]. In addition, the pharmacist must also often deal with patients belonging to different ethnic, cultural, linguistic, and religious backgrounds, including potential eating habits-related illnesses (e.g., rickets and osteomalacia due to malnutrition or dietary deficiencies) [52], specific religious-related food prohibitions or traditions (e.g., pork and Ramadan for Muslims), spoken or written language barriers, etc. The pharmacists, therefore, must firstly understand the needs of each patient and adapt the message to the recipient [3,43]. Health provider attitude sometimes can be an obstacle to effective patient-centered communication, hence the pharmacist “must maintain a high level of humility about their scientific knowledge so that the knowledge of the patient can be recognized” [43,53].

After this preliminary “analyzing” phase, when the patient is finally comfortable in asking for health advice, the pharmacist can finally assess the health problem and then start a tailored interpersonal dialogue which leads to bestowing advice and recommendations about pharmacological treatment, drug intake modalities and possible adverse effects, and also suggesting the patient, if necessary, ask for further medical advice [3,27].

Given the above situation and the complications which can characterize the pharmacist-patient communication, it is absolutely necessary that the professional pursues an active approach in relation to the patient providing information and advice, using a verbal as well as a written approach and, if necessary, collaborating with other health professionals or agencies [45].

A potential support to the pharmacist-patient interaction could be resorting to the late 70s Prochaska and DiClemente Trans-Theoretical Model (TTM) of intentional behavioral change, also known as the Stages of Change model, developed by drawing on various disciplines and tested in a wide range of behaviors [54]. Regarding the implementation of TTM in the community pharmacy context, we can distinguish five main scenarios: 1. *Pre-contemplation*: while the patient, satisfied with his/her “*modus vivendi*” (i.e., lifestyle) is not inclined to change behavior, the pharmacist, without expecting to success, can try to persuade him/her to modify the “bad habit”; 2. *Contemplation*: the patient reflects on the possibility of changing the bad habit, while the pharmacist can help him/her in developing a possible plan to implement these changes; 3. *Preparation*: the patient took the decision to change and the pharmacist helps him/her to build a plan and set goals; 4. *Action*: the patient implemented the change and the pharmacist takes a supportive approach, establishing a permanent relationship; 5. *Maintenance*: while the patient is training to avoid any relapse, the pharmacist can continue to play the supportive role, providing encouragement and positive feedback [3].

In the everyday practice, TTM allows the pharmacist to properly check the advancement of a patient’s status, tailoring the medical advice and providing the most accurate health care possible. Caponnetto et al. [55] showed how TTM can be successful with the trained community pharmacists’ role in patient-centered counseling on smoking cessation therapies (e.g., OTC nicotine replacement therapy (NRT)). The study involved 42 community pharmacies that attended a conference focused on the US Public Health Services 2008 “Clinical Practice Guidelines for Treating Tobacco Use and Dependence.” Then 21 pharmacies were randomly assigned to the control group and 21 to the intervention group. The intervention group attended a specific anti-smoking training (based on TTM) that led pharmacists to be more effective and skilled in anti-smoking counseling. The 124 smokers in the intervention group who asked to start a smoking cessation path were offered a patient information sheet, in which pharmacists’ attendance to the specific training was documented. On the other hand, the 63 smokers in the control group were informed that the pharmacists attended only the conference. With the aim of involving patients, the pharmacists resorted to a confidential record (containing any product supplied, questions raised, advice given, etc.), a plan of follow-up visits, and telephone call reminders. At week 24, the study underlined higher effectiveness of community pharmacists trained in TTM on smoking cessation in helping smokers to quit compared to the control group (“the intervention group smoked a mean of 22.4 cigarettes per day at recruitment versus a mean of 7.5 cigarettes per day at week 24; the control group smoked a mean of 17.3 cigarettes per day at recruitment and 12.5 cigarettes per day at week 24”) [55].

TTM framework has a significant role in helping health providers to understand human behavior changes in order to give social support and accessible health care services, to strengthen the patient-treatment confidentiality, and to face the maintenance phase. In this regard, in the specific case of treating alcohol dependency, Yamarthi et al. [56] underlined that addressing the patient’s issues only using a biological and scientific approach is not sufficient, but it’s necessary to use a more holistic one (e.g., helping patients in following a unique “multimodal biopsychosocial” treatment that includes cognitive-behavioral components) [56].

An interesting example of a practical application encompassing all of the above necessities and dimensions is given by the implementation of the Alberta (Canada) Comprehensive Annual Care Plans (CACPs), as reported by the 2019 Schindel et al. comprehensive study [9]. Resulting from a pharmacist-patient collaboration, the CACPs aim at documenting the tailored health care path that each patient undertakes and that can be later shared with other health professionals. By turning to pharmacists for “customized” health plans, patients gained first and foremost consistent advantages in terms of time saved compared to the waiting time for specialist appointments. Moreover, “the community pharmacy environment was more conducive to conversations about health and concerns” due to the less formal nature of conversation, thus reflecting the “trust and confidence in the relationships built between patients and the pharmacists.” Particularly interesting was the fact that, thanks to this family-like environment, patients were keener to ask further and more detailed questions to pharmacists regarding health conditions not necessarily linked to their CACP. After a first phase of trust-building in which patients’ willingness to collaborate played a central role, the CACP constant monitoring action reinforced and improved the pharmacist-patient relationship, which eventually led the latter to consider the former as a “secret doctor.” As the study reports, “patients began to see pharmacists as primary health care providers who could do more than «fill prescriptions»” and, at the same time, “pharmacists derived greater satisfaction with their work” feeling that “they were «contributing more» to patient care and primary health care, that they were staying «more current», and that they enjoyed having «people depend on [them]»” [9].

### 3.1. Quality Communication

“This is an important duty, for one can hardly imagine anybody who could be more dangerous to the public than a pharmacist who is liable to make errors in dispensing what may be dangerous drugs”, Sir Gordon Willmer, Chairman of the Pharmaceutical Society’s Statutory Committee, Pharmaceutical Journal [3].

We made the case for the need for a deeper and stronger pharmacist-patient interaction which must be ultimately based on a bilateral, communication-based relationship. At this point, a further necessary step consists in the assessment of the pharmacist-patient communication quality, a fundamental element to guarantee the success of the above-mentioned communication-based relationship. In fact, improper and inaccurate communication, together with the aforementioned self-medication and low patient “health literacy” issues, can easily lead to misunderstandings of medical recommendation, deviations from prescribed treatment regimen, drug abuse, etc. [3].

**Figure 2:**
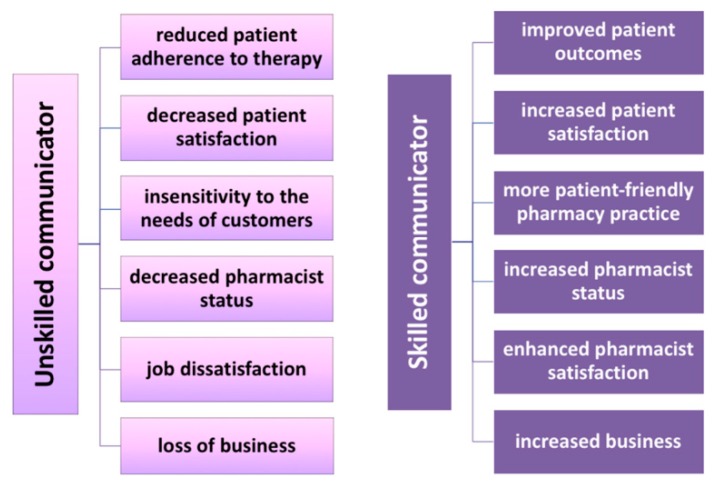
Consequences of the pharmacist as an unskilled or skilled communicator.

In this regard, Ley and Llewellyn in 1995 [57] stressed the importance of linking together the elements of understanding, recall, satisfaction, and adherence with regard to information quality received by the patient. Adherence in particular refers to a more active involvement of the patient in the therapy, so that he/she is ready and more capable of collaborating with the pharmacist and, in general, with health sector professionals in planning and implementing the treatment regimen [58]. The solution therefore consists of improving the pharmacist’s communication skills through the simplification and clarification of the medical language in order for the patient to properly understand the advice received. The 1996 research conducted by Dickson et al. [59] further underlined the importance of communication quality between the healthcare professional and the patient, pinpointing five main consequences of low-quality communication (Figure 2): 1. overload or over-complexity of information: the patient does not understand or forgets the information received and is reluctant to raise clarifying questions; 2. relational shortcomings (e.g., inability to create a visual contact or to establish an empathic dialogue): the patient is dissatisfied with the advice, the information received, and the ways of managing the dialogue; 3 and 4: loss of patient satisfaction and adherence to the prescribed therapy due to the shortcomings of points 1 and 2; 5. inattention to the patient’s psychological needs (e.g., the professional is insensitive to the patient’s mood, opinions, needs and doubts, and cannot tune to the non-verbal signals sent by the patient).

Academic literature has highlighted in general eleven areas of key communication skills [60]. They are: 1. *Opening*: greeting the patient possibly by name, searching for it in the prescription; 2. *Building the rapport*: preserving the confidentiality of the dialogue by implementing the conversation in an appropriate location, being helpful and available/accessible, having good manners, showing involvement and sincere concern, offering reassurance, meeting the needs of the patient; 3. *Active listening*: showing empathy, being focused and objective, not stereotyping, and encouraging the patient; 4. *Non-verbal communication*: using eye contact, paying attention to the body language, being close to the patient through an open posture, using gestures, smiling, nodding, and illustrating; 5. *Explaining*: giving motivated instructions and explanations with the aim of reassuring, providing clear information through simplified scientific and medical language to assure a proper and easy implementation and understanding, repeating and emphasizing, using analogies or examples, and accompanying the verbal message with a written or visual aid, if necessary; 6. *Questioning*: using open questions to involve the patient, asking about other drug intakes, about possible symptomatology, making a real exploration of the situation by collecting meticulous details and showing interest; 7. *Suggesting/advising*: providing professional/personal opinions to direct the patient towards a specific action/choice rather than another; 8. *Assertiveness*: politely expressing personal thoughts and feelings and strengthening the credibility by recommending to the patient, if necessary, to ask for further advice from other health professionals, 9. *Self-disclosure*: sharing personal experiences with the patient to reassure him/her or showing to be involved in the situation that afflicts him/her; 10. *Persuading*: stimulating the moral duty of each individual or raising concern to push the patient to modify certain bad habits; 11. *Closing*: being kind, summarizing the main points discussed during the patient consultation and making sure to receive positive feedback about it, creating a link for potential future interactions by stimulating the person to return to the pharmacy, thanking and praising the patient, beginning the final phase of interaction by physically moving from the counter, and finally, using closing indicators to complete the conversation [51,60].

Ineffective communication can cause consequences affecting the patient, but also the pharmacist, such as greater concern among patients leading to a regression of the pharmacist’s professional status, to job dissatisfaction, to a loss of customers and, therefore, to a loss of business [59]. On the contrary, if the pharmacist was a skilled communicator, communication with the patient would be more effective and the final result would be positive for both, in particular in terms of patients’ satisfaction since they would have a more positive view of pharmacy, considering it as a practice open to their specific needs. The pharmacist would acquire greater personal satisfaction and self-esteem, would see an increase in the clientele, and would obtain a commercial advantage to strengthen his/her business (Figure 2) [59].

### 3.2. Health Literacy and Communication Training Program

Health education and communication are fundamental elements in the context of health promotion. The efficient supply and appropriate use of health services by pharmacists in community pharmacies are considered important factors in determining the patient’s health status. The phase of health promotion is influenced by all of those personal, social, and structural factors that can be modified through the application of specific strategies and models, with the aim of making health promotion effective and correct [61]. Specifically, “health literacy” refers to those personal, cognitive, and social skills that determine the ability of individuals to obtain, understand, and use basic health information, and all services aimed at promoting and maintaining a healthy lifestyle [61,62,63,64].

In this regard, Freebody and Luke [65] distinguish three types of literacy levels respectively characterized by the possession of: 1. basic functionalities sufficient for reading and writing, necessary to be able to face everyday situations; 2. communicative/interactive literacy consisting of more advanced cognitive and literacy skills that, together with social skills, allow for more active participation in daily activities; 3. critical literacy, namely those more advanced cognitive abilities which, together with social skills, can be applied to analyze information critically [65]. Such classification qualifies interactive and critical literacy as essential elements constituting health literacy in the context of practice in the community pharmacy. Hence, the pharmacist, who by definition possesses these two types of literacy, should not limit himself to reading brochures and leaflets, but should be able to provide advice aimed at improving people’s access to health information, as well as at enhancing their ability to use it in the most effective way [61].

Concerning Europe, Veenker and Paans estimated that in EU countries 10%–30% of the population doesn’t have a sufficient level of health literacy and, as a consequence, suffers from higher morbidity and mortality and, at the same time, health services utilization is higher and treatment outcomes are unsatisfactory. Moreover, 12% of Europeans don’t have adequate health literacy competence and for 35%, these competences get even more problematic [64]. Furthermore, the processing of the results of the first European comparative survey on health literacy in populations conducted by Sorensen et al. [66] has shown that, although with the distribution of levels that differed substantially across countries, at least 1 in 10 (12%) of respondents showed insufficient health literacy and almost 1 in 2 (47%) had limited (insufficient or problematic) health literacy. Considering that patients with lower health literacy are more likely to misunderstand the instructions provided on the label of a drug compared to those with marginal or adequate literacy [67,68], many people in Europe (in particular, the poor and the elderly) therefore have levels of health literacy below those necessary for the correct and effective understanding of information related to the drug [64].

**Figure 3:**
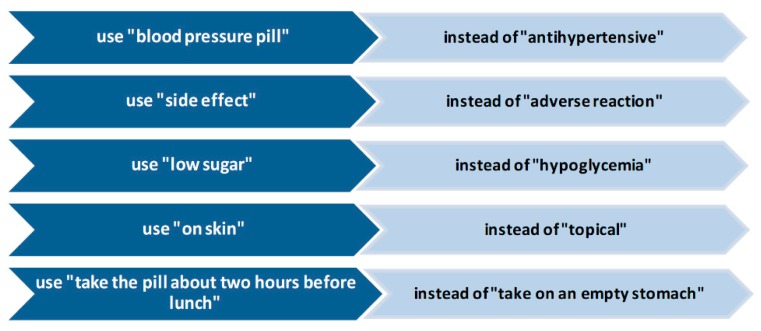
What a pharmacist could say instead of using medical terminology [62].

An additional problem is given by the fact that most individuals who fall into the above-mentioned categories are often able to mask their cognitive deficits. The pharmacist must therefore be able to understand these situations by intercepting some specific signals sent by the patient, such as certain phrases and/or questions (e.g., “I forgot my glasses,” “let me take this home,” “I’ll read it later”) [62]. Since this is not always possible, it would be more appropriate to adopt a simplified universal language for all contexts and situations, taking into consideration that the communication guiding points for pharmacists should be the “*making sense*” and the “*supporting autonomy in making choices*” (Figure 3) [64]. In this sense, Kripalani and Jacobson [62] suggest a scheme of strategies that the pharmacist could apply daily during the phase of interaction with the patient, hence allowing him/her to greatly improve his/her communication. This scheme would consist of five key points: 1. to give clarifications and explanations in plain non-medical language, making a great effort in avoiding the use of medical jargon; 2. to focus on the message to provide and to recall it several times during the conversation; 3. to use the “teach back” tactic, also called “show me” (retroactively instructing), to check if the patient has understood the message well (e.g., the pharmacist could ask the patient to repeat what he said during the consultation, instead of asking directly if he understood); 4. to solicit the patient effectively with questions such as asking “what questions do you have to ask me?” instead of “do you have any questions?” or “questions?”; 5. to use support material, such as simply and concisely written information, together with the verbal materials, which are even better if they are accompanied by images and illustrations [62]. In general, the points mentioned should move towards an ever-increasing involvement of the patient in the communication process.

What seems of fundamental significance is the systematic consequence of a widespread high level of health literacy, which clearly exceeds the boundary of mere cultural aspect. As Veenker and Paans report, it is important to stress how “there is an association between the levels of health literacy and the self-assessed health status in the population: higher levels of health literacy go hand in hand with higher self-assessed health status, while low health literacy is associated with lower perceived health status”. A high (or just adequate) level of health literacy generates a higher self-awareness regarding one’s own health status. This increased awareness leads to better and more effective self-medication and self-management. Ultimately, this greater autonomy will lead to a lowered impact of household drug expenses and of the “weight” that each individual (in particular elderly people) exercise on national health systems [64].

## 4. The Educational System and the Future

The above-mentioned necessity of simplifying the medical terminology into a more confidential and accessible language can be quite complicated for the pharmacist. In order to guarantee students have the right skills necessary to conduct a clear and comprehensive dialogue with the patient, tutors should include educational methods which stimulate students to carefully reflect on the “language register” used and, if necessary, lead them to learn new and more appropriate terminology. This should be then integrated into the practice of counseling sessions, giving students the opportunity to learn how to put into practice the acquired theoretical communication skills. Students need training to evolve into health care professionals with their own communication styles that could be then effective for counseling and in all the other phases characterizing the pharmacist-patient interaction (listening, questioning, etc.) in patient-centered care services [69]. In this regard, Wallman et al. revealed that in pharmacy education, good training occurs with patient-focused communication activities (e.g., learning interviewing techniques, patient counseling, or public health promotion) [28]. Other literature describes the introduction of student pharmacist assessments on oral patient communication (e.g., structured exam, pre/post evaluations) [70,71,72,73,74].

In 2007 the US Accreditation Council for Pharmacy Education (ACPE) created guidelines (revised in 2016) [75] setting standards that new graduate pharmacists should follow in order to cover and fulfill their responsibility in communicating with patients. It has proven to add value for the study path of pharmacy to include training courses based on Introductory Pharmacy Practice Experiences (IPPEs) and Advanced Pharmacy Practice Experiences (APPEs) in the training curriculum. This project therefore gave students the opportunity to deepen their knowledge in order to optimally perform the additional services offered today in the pharmacies, together with drug dispensation and advice-giving. The APPEs also gave students the possibility of practically developing the communication skills studied at an academic level and being able to build an interpersonal relationship with the patient [51]. IPPEs and APPEs programs are included in pharmacy school curricula in the US [76], as well as in Australia, where a training course in social pharmacy and the acquisition of communication skills has become mandatory in order to be professionally qualified [28], and the same is required in Canada [77].

In the EU context, in Denmark the study of social pharmacy is a compulsory subject within the academic curriculum, while in Finland, the bachelor’s degree in pharmacy includes in its curriculum the study of how to set up proper communication, from counseling to patient education to health. This path also includes communication techniques (specifically to establish the interaction with the patient) and the study of foreign languages [28,78].

There is a lack of literature on pharmacy patient-centered communication and the few existing examples applied to pharmacy practice are derived from those developed for physicians [43,79,80], such as the Calgary-Cambridge guide (CCG) [81] and the Four Habits model (FHM) [43,82].

Applying the CCG (an evidence-based model) enables pharmacists to tailor medical consultation through the improvement of 71 communication skills and behaviors [81]. The FHM instead includes 23 elements of health professional communication behaviors categorized into four “habits” [83,84,85]. The outcomings from the FHM application in the pharmacy field raised the need for the ideation of a new instrument, specific to student pharmacist-patient communication skills, able to both measure communication and to assess the pharmacist-patient relationship. This framework, called the Patient-centered Communication Tools (PaCT), includes 23 skills organized into five general “tools” to be taught to students at an academic level [43,74].

An interesting example is the development of a set of practice standards for communication skills within the actions implemented in 2012 by the UK National Modernizing Pharmacy Careers (MPC) program. The main purpose was to individuate “the standard of knowledge, skills and behaviours expected of all pharmacy professionals in order for them to carry out effective patient-centred consultations”. The initiative was integrated by the national Consultation Skills for Pharmacy Practice Programme (later transferred to Health Education England, HEE) and by the possibility of consulting an online national learning portal (www.consultationskillsforpharmacy.com) where pharmacists can self-assess their own level of communication skills. In general, the program balances theoretical and practical tools for pharmacists to enhance their communication skills by providing six thematic “routes” that the professionals can undertake to perfect his/her skills and which are related to: 1. medicines optimization and adherence; 2. effective communication fundamentals and patient history; 3. history and background to consultation models, and theory and consultation styles affecting practice; 4. effective consultation skills, shared decision-making, and tools supporting practice; 5. health coaching and motivational interviewing to support behavioral change; 6. action planning to move your practice forward [86].

With regard to what has been said so far, for example in the European Nordic context (Norway), community pharmacists, the regulatory setting, and the undergraduate academic education have responded to the shift in the pharmacist’s role towards the increased focus on the communicative interrelations with the patient. The pharmacy practice is essential in developing and training communication skills for the patient encounter. In spite of that, an experiential training method is not provided in all schools, it is often concentrated approximately in the last part of the five years of academic education curricula, and it consists of a number of hours that varies from six to 92. Therefore, the regulations are not supporting the pharmacist role as they should. Among the goals of the course developed by leaders in the Nordic pharmacy, the context is to outline and improve communication skills in order to both enhance the community pharmacist training and strengthen his/her professional role [87].

Considering all of the above, universities should offer even more specifically tailored pre- and post-graduate training for those students willing to work in community pharmacies. By doing so, the new graduates would be allowed to achieve a specialization specifically taught for the practice of the profession in the community pharmacy, including appropriate training on communication and social skills [78]. Moreover, apart from the communication-based training, future community pharmacists need to be prepared on many other fundamental topics ranging from survey consulting to monitoring adverse reactions, from prescribing practices to pharmacy management, from computer data analysis to health legislation and economics, etc. [78]. Thanks to these changes in the educational field, the pharmacist would learn the right way to give information on medicines and would practice the development of communication skills essential to ensuring the professional future of the pharmacist role in community pharmacy.

## 5. Conclusions

Nowadays, the pharmacist’s profession in the community pharmacy is not highly regarded since the commercial vision of the profession created for pharmacists includes the image of the shopkeeper. Some pharmacists in some countries are no longer involved in dispensing, but only in providing the drug and giving advice [3]. In spite of their notable training, community pharmacists are the only health professionals who are not primarily rewarded for delivering health care. Community pharmacists may play a role in reducing ADEs and in increasing medication adherence, which in turn could help in lowering unnecessary provider visits and hospitalizations while reinforcing integrated primary care delivery across the health system [14]. Furthermore, the perception of the pharmaceutical profession in the community pharmacy is increasingly at risk, especially because of the impact of the Internet on society. By resorting to these means of artificial communication, personal communication between the pharmacist and the patient has been reduced or has even disappeared.

Since one of the key roles of the pharmacist in the community pharmacy is focused on counseling, communication is essential for the pharmacist in order to fulfill his primary ethical duty. Communication is also indispensable for the patient in order to receive all of the necessary information on the use of a given medicine in an interactive, direct, clear, and detailed manner, and to acquire the knowledge to obtain the maximum benefit from the therapy to be performed. Communication is therefore the key and essential element in order to build a solid interpersonal relationship with the patient in order to retain him/her, to make the consultancy process effective, and to strengthen the future of the pharmacist profession in the community pharmacy.

In a future perspective, to revitalize the community pharmacist role and to improve his/her specific patient-centered communication skills and health literacy, it is essential to implement adequate and common changes in the academic curricula, which need to be regularly monitored and up-to-date [78,88]. A more widespread introduction of in-depth study and professional training in behavioral, communication, educational, and sociological methodologies and techniques would guarantee even more effectiveness in pharmaceutical counseling practices. Starting from the practical experiences analyzed (in particular, PaCT and MPC), learning a common, standard patient-centered communication method could allow the pharmacist to use a simpler, more direct, and clear language in his/her daily practice.
